# Rapid Antibacterial Assessments for Plastic and Textile Materials Against *Escherichia coli*

**DOI:** 10.3390/antibiotics13121156

**Published:** 2024-12-02

**Authors:** Anson M. Y. Luk, Adrian M. H. Luk, Jiachi Amber Chiou, Man-Yi Ho, Chi-Man Ngai, Chi-Wai Kan

**Affiliations:** 1 School of Fashion and Textiles, The Hong Kong Polytechnic University, Hung Hom, Kowloon, Hong Kong SAR, China; anson-m-y.luk@polyu.edu.hk (A.M.Y.L.); man.yi.ho@connect.polyu.hk (M.-Y.H.); 2 Immune Materials Limited, Room 05, Unit 107-109, 1/F, 9 Science Park West Avenue, Hong Kong Science Park, Pak Shek Kok, N.T., Hong Kong SAR, China; spikengai@immunematerials.com; 3 Department of Food Science and Nutrition, The Hong Kong Polytechnic University, Hung Hom, Kowloon, Hong Kong SAR, China; adrian-mh.luk@polyu.edu.hk (A.M.H.L.); jiachi.amber.chiou@polyu.edu.hk (J.A.C.)

**Keywords:** AATCC TM 100, antibacterial, *Escherichia coli*, ISO 22196, MicroSnap, rapid tests

## Abstract

**Background**: Standard test methods for evaluating the antibacterial performance of plastic (non-porous) and textile (porous) materials are accurate and reliable, but completing a standard assessment generally requires at least several days to a week. Well-trained and experienced technicians are also required to conduct the standard tests consistently and analyse the samples and test results systemically. These costs are often not favourable for the performance assurance of antimicrobial products in industrial production, nor for meeting the fast-return demands in research and development of antimicrobial materials nowadays. **Methods**: In this study, “Rapid Tests” are developed to evaluate the antibacterial activities of plastic and textile materials. **Results**: The assessment results from Rapid Tests for plastics and textiles are highly correlated to those from the ISO 22196 and the AATCC Test Method 100, respectively, whereas the evaluation operation can be completed within one day. Based on bioluminescence technology, colony-forming units of *E. coli* from the inoculated specimens are determined via luminometry. Antibacterial efficacy of the treated plastic and textile samples can be examined effectively. **Conclusions**: By analysing antimicrobial artificial leather samples composed of hydrophilic polyurethane polymer using Rapid Tests for plastics and textiles, the applicability and scope of these tests were remarkedly recognised and verified.

## 1. Introduction

Different effective methods of evaluating the antibacterial performances of plastic (non-porous) and textile (porous) materials are standardised. The ISO 22196 Test [1] and the AATCC Test Method 100 (AATCC TM 100) [2] are the standard quantitative tests which are commonly employed for assessing the antibacterial efficacies of plastic and textile samples, respectively. The principle of both standard tests is actually similar. An inoculated bacterial solution is washed out with sterile neutralising solution to collect the remaining bacteria for cell culture in a serial dilution manner [3,4]. Determination of the colony-forming unit (CFU) by counting the colonies formed on the cultured agar plates with the bacterial solution collected from the inoculated samples is reliable and accurate; however, the span of conducting such complete standard tests generally requires a few days to a week by trained and experienced technicians. It is not considered a satisfactory and cost-effective practice for developing antibacterial prototypes in R&D and executing quality assurance in commercial production of antimicrobial products.

In this study, rapid assessment methods, termed as “Rapid Tests”, are developed for preliminary antibacterial testing of non-porous and porous samples, i.e., plastic and textile specimens, respectively. The assessment results of plastic and textile samples by Rapid Tests are compared with those of the ISO 22196 and the AATCC TM 100, respectively, in order to verify the reliability and consistency of the Rapid Tests. The test inoculum, *Escherichia coli* (*E. coli*), is a common Gram-negative bacterium for antibacterial tests. Although *E. coli* is a commensal organism of the vertebrate gut microbiota, it is widely recognised as an opportunistic pathogen of humans and animals [5]. As reviewed by Denamur et al. [6], *E. coli* strains can cause both intestinal and extraintestinal diseases. Intestinal ones include various forms of diarrhoea, such as haemolytic uraemic syndrome (HUS). Extraintestinal conditions encompass urinary tract infections (UTIs), diverse intra-abdominal, pulmonary, skin and soft tissue infections, newborn meningitis (NBM), and bacteraemia. These common infections tend to result in high morbidity by renal failure in HUS in children and neurologic sequelae in NBM, as well as high mortality by bacteraemia with about a 15% fatality rate. For instance, outbreaks of HUS epidemics often occur worldwide, posing significant health threats to children under five years of age and the elderly over 60 [7,8,9]. The World Health Organisation [10] has also raised increasing concern about antibiotic resistance in *E. coli*, which is ranked third on the list of 12 antibiotic- resistant “priority pathogens”. Therefore, the evaluation of antibacterial prototypes or samples against *E. coli* is an essential and fundamental practice.

The principle of Rapid Tests is based on bioluminescence technology [11,12] to detect and enumerate *E. coli* strain remaining after inoculation of a test specimen within 6 h, as designed by the test equipment and system. Rapid Tests are developed with Hygiena’s MicroSnap Coliform & *E. coli* Enrichment Device, a MicroSnap *E. coli* Detection Device, and an EnSURE V2 luminometer for evaluations. Such a test system is certified by the Association of Official Agricultural Chemists (AOAC) Research Institute and has obtained AOAC Performance Tested Certificate No. 071302; the validation of the MicroSnap *E. coli* test system by Hygiena International was recorded in Journal of AOAC International [13]. *E. coli* inoculum is collected and cultured in the Enrichment Device in the presence of a proprietary nutrient growth medium with an inducer of β-glucuronidase enzyme. As the β-glucuronidase accumulates within the *E. coli* bacteria and does not export into the growth medium, the concentration of enzyme is proportional to the number of bacteria. After incubation for a certain period of time, an aliquot extracted from the Enrichment Device is transferred into the Detection Device, which contains a lysis reagent with adenosine triphosphate and β-glucuronidase substrate. By adding a controlled amount of luciferase reagent using the unique setup of the device, the quantity of β-glucuronidase interacting with the bioluminogenic substrates can be measured by the luminometer to obtain the relative light unit (RLU). There is a strong proportional correlation between the emitted light signal, the concentration of the enzyme, and the bacterial count [13,14].

Compared to traditional standard tests, the newly developed Rapid Tests are relatively promising test methods with a short return time and lower operation cost for in-house assessments [15,16]. The ISO 22196, the AATCC TM 100, and other common standard methods cannot be replaced by Rapid Tests owing to their superior accuracy and more complete scientific support, especially when conducted by third-party testing laboratories and well-trained technicians to ensure the interests of manufacturers, suppliers, and customers. Furthermore, since Rapid Tests are designed to determine the bacterial counts based on the concentrations of β-glucuronidase that has reacted with bioluminogenic substrates, Rapid Tests make it possible to calculate the number of *E. coli* that are ever and still viable after inoculation and collection by quantifying the accumulated β-glucuronidase during incubation. This can be considered as one of the technological strengths of Rapid Tests when compared with traditional standard methods. Some bacteria can still survive and be infectious during a daylong incubation period after inoculation and collection, but they will not form colonies in the plate methods employed in the standard tests. As viable *E. coli* are also counted in Rapid Tests, over-estimation of the viable bacterial counts can occur when contrasted with standard test results [15]. On the other hand, the evaluation of minimum inhibitory concentration (MIC) by Rapid Tests is more accurate and convenient than standard test methods. The inhibited but viable *E. coli* can be detected effectively in Rapid Tests instead of using the plate methods of standard tests, where the *E. coli* cannot be cultured well to form accurate colonies owing to the aliquots containing unavoidable and non-negligible amounts of antimicrobial agents. MIC is often estimated by traditional plate methods [17]. Thus, Rapid Tests are a cost-effective way to evaluate the MIC of antimicrobial plastic and textile materials to be developed.

To our knowledge, the development of Rapid Tests or similar testing for antibacterial assessments of plastic and textile materials has been rarely reported. Hong and Kim [18] developed a software to count the colonies formed in the antibacterial test used in textile materials, the AATCC TM 100, to reduce the time of manual counting, and to increase the accuracy and reproducibility of bacterial counting via image analysis. Xia et al. [19] reported the evaluation of *E. coli* and *S. aureus* activities via spectrophotometry instead of plate counting in order to save time, but also to ensure the use of sufficiently active bacteria for subsequent antibacterial textile assessments; more significant and reliable results can be obtained to avoid the time spent on re-assessments. Other research on antibacterial tests focuses on comparative studies on different (standard) test methods involving extensive incubation and bacterial colony culture [4,20,21,22,23], which are the most duration-heavy processes in antibacterial tests. Even though a new standard method, the ISO 7581:2023, has been established for non-porous materials to address the deficiencies of the ISO 22196 [24], prolonged incubation, bacterial colony culture, and plate counting are still involved. It is believed that the Rapid Tests offer a simpler and more efficient, but still reliable approach to preliminarily examine materials with antimicrobial treatments.

## 2. Results and Discussion

### 2.1. Antibacterial Tests of Non-Porous Samples

The antibacterial activities of silicone rubber, high-density polyethylene (HDPE), and three-dimensional-printed (3D-printed) photo-polymerised plastics were evaluated using the ISO 22196:2011 [1] test and the Rapid Test for plastics; the results are summarised in Table 1 and Table 2, respectively.

The antibacterial activities against *E. coli* of the antimicrobial plastic samples are significantly demonstrated by both test methods. Contact-killing and 24 h inhibition towards *E. coli* were exhibited by these samples in ISO 22196 tests. On the other hand, the plastic samples were inoculated with two concentrations of *E. coli* solution for 10 min in the Rapid Test. Such inoculated bacteria collected from the surfaces of antimicrobial plastic samples could not be cultured well in the Enrichment Devices. Limited β-glucuronidase were produced by such negligible amount of *E. coli* to react with the bioluminogenic substrates in the Detection Devices. As a result, very weak signals of emitted light were measured by the EnSURE V2 luminometer, quantifying that such low colony-forming units (CFUs) of *E. coli* remained. In contrast, the CFU/swab values of the *E. coli* collected from the pristine substrates of silicone rubber, HDPE, and 3D-printed plastics indicated large amounts of *E. coli* grown on the surfaces of the pristine plastic materials. The antibacterial plastic samples demonstrated over 99% efficacy against *E. coli* (see Table 2). Such strong effects towards the test inoculums of the high and low concentrations tend to represent the promising performances of antimicrobial plastic materials. In contrast to the ISO 22196 tests in Table 1, as Rapid Tests can count the *E. coli* that remain and are still viable after inoculation and collection, the results of the antimicrobial specimens were not all recorded as 0 in Table 2. This indicates that some *E. coli* were still viable before being assessed by the Detection Device; however, the maximum bacterial counts among those antimicrobial specimens did not exceed 35 (for the 3D-printed rigid plastic specimen inoculated with 1:10^8^ *E. coli*). This markedly demonstrates the potent bacterial reduction performance, over 99%, of the antimicrobial specimens.

“Direct pipetting” and “swabbing” are two reference setups to measure the activities of *E. coli* directly inoculated to the Enrichment Device and collected from the inoculated inert surface (clean Petri dish) via the swabs of the Enrichment Device, respectively. They are able to validate and monitor the reliability and accuracy of the Rapid Test each time. In normal cases, the RLU value of a pristine sample is higher than that of the direct pipetting and swabbing references. This typically implies that the inoculated *E. coli* are sufficiently active for the test and prone to growing on the untreated plastic surfaces, although Hygiena International tends to cap the CFU/swab value at 1.00 × 10^4^ when the RLU value exceeds 300 units. This phenomenon is actually correlated to the ISO 22916 test that the CFUs of *E. coli* grown on the pristine sample surfaces after incubating for 24 h should be larger than that for 0 h (see Table 1), owing to the formations of biofilm on plastic surfaces and particular bacteria–plastic interactions [25]. Silicone rubber, HDPE, and 3D-printed rigid and tough plastics can represent four different plastic surfaces as they have various surface properties in terms of roughness, hardness, density, porosity, and hydrophobicity (hydrophilicity). The bacterial adhesion and growth are directly influenced by these surface properties of plastics [26]; it can be further studied and evaluated using Rapid Tests.

### 2.2. Antibacterial Tests of Textile Samples

Cotton fabrics finished with a cationic antimicrobial agent (CAA) were examined using the AATCC TM 100-2019 [2] and Rapid Tests developed for textile materials. The test results by AATCC TM 100-2019 are listed in Table 3; the ones by Rapid Tests conducted using volume and dilution approaches are presented in Table 4 and Table 5, respectively.

In AATCC TM-100, cotton fabrics treated with 0.5% and 1.5% (*w*/*w*) CAA demonstrated satisfactory antibacterial effects against *E. coli* in terms of contact-killing and 24 h inhibition. These results are correlated to the results of Rapid Test in Table 4. Various volumes of *E. coli* in three concentrations were inoculated to the test specimens in the volume approach of the Rapid Test for textiles. Apart from the results from the setups inoculated with 10.1 and 20.1 µL of 1:10^6^ *E. coli* solutions, the other setups revealed significant differences in CFUs between the untreated and treated cotton specimens, thus confirming the potent antibacterial activity against *E. coli* of the cotton textiles functionalised with the CAA. As explained in Section 2.1, although the antimicrobial cottons exhibited no bacterial counts in the AATCC TM 100, some of the samples did not exhibit RLU results of 0 in Rapid Tests owing to the inclusion of the ever or still viable *E. coli* after inoculation and collection, which did not exceed the bacterial counts of 15 in Table 4 and Table 5. The more significant antibacterial efficacy of textiles compared to plastics is probably attributable to the larger surface area of contact between fabric sample and the inoculum, leading to more effective bacterial killing.

The less significant results obtained by using 10.1 and 20.1 µL of 1:10^6^ *E. coli* inoculum are attributable to the amounts of inoculum not being adequate for growing the *E. coli* in the Enrichment Devices after 6 h. Comparing the reference setups, i.e., direct pipetting to saline solution, for 1:10^6^ *E. coli* inoculum, the decline of CFUs from 30.1 µL to 20.1 and 10.1 µL is convincing evidence. Even though a considerable amount of *E. coli* bacteria could still be cultured in the reference setups of 10.1 and 20.1 µL of 1:10^6^ inoculum, the CFUs obtained from the pristine cotton fabric setups inoculated with 10.1 and 20.1 µL of 1:10^6^ bacterial solution were exceptionally low. These false positive antibacterial results clearly demonstrate that using too low a concentration and volume of *E. coli* bacterial solution (threshold ≤ 30.1 µL of 1:10^6^ inoculum in this setup) in the test system renders the antibacterial test insignificant.

Sterile 0.85% (*w*/*v*) saline solution was utilised to rinse and extract the *E. coli* inoculum from textile specimens in the Rapid Test. In another trial of the Rapid Test for textiles, the saline aliquot was further diluted using the same saline solution via 10-fold serial dilution, which is a common cell-culture practice in standard antibacterial tests [27], to perform the Rapid Test using a dilution approach. The trial results are presented in Table 5. Only the test setup of 1:10^8^ *E. coli* inoculum diluted 10 times could still exhibit significantly different results between the untreated and treated specimens. Diluting 1:10^8^ inoculum 100 times and other concentrations of *E. coli* inoculum tended to demonstrate the least significant results between the untreated and treated specimens, and even in some direct pipetting references. It is considered that the amounts of *E. coli* inoculums extracted and collected were not sufficient after serial dilution for enrichment. Therefore, the dilution approach is less practical than the volume approach for Rapid Tests. A greater consumption of sterile saline solution in the dilution approach should also be noted.

### 2.3. Case Study About Assessing Highly Hydrophilic Polyurethane Plastic Samples by Rapid Tests

Artificial leathers are commonly prepared by coating or laminating textile fabrics with hydrophobic polymers, such as polyurethane (PU), polyvinyl chloride (PVC), and silicone rubber to mimic the tactile feel, surface textures, and properties of genuine leathers [28]. In this study, polyester fabric was coated with a thin layer of PU doped with 1 to 2% CAA to fabricate artificial leather samples with antibacterial functions. It is generally accepted that the surface of artificial leathers features superior easy-care and repellent properties compared to that of genuine leathers [29,30]. By preliminary observation, the PU-coated side of the artificial leather samples looked like a plastic layer with certain water-proof properties. Antibacterial efficacies against *E. coli* of the samples were evaluated using the Rapid Test for plastics; the results are summarised in Table 6. The reference setups of direct pipetting and swabbing indicated a strong activity of *E. coli* inoculum in the two concentrations. Artificial leather samples treated with CAA also exhibited effective biocidal actions towards the *E. coli*. However, it is unusual that the pristine artificial leather also showed comparable antibacterial efficacy to the treated samples. This false positive antibacterial result can be attributed to a less appropriate test method and the biotoxicity issue of the PU coating material.

**Figure 1:**
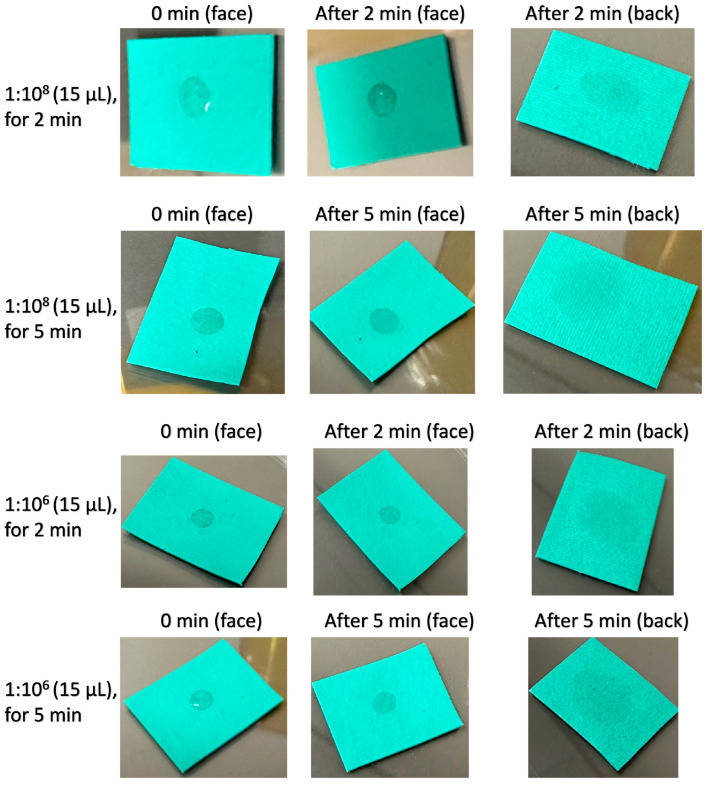
The photos of face and back sides of artificial leather samples (prepared with polyester textile face coated with polyurethane polymer), after the face sides were inoculated with 15 µL of *E. coli* solutions in 1:10^6^ and 1:10^8^ at 0, 2, and 5 min (The more obvious the watermark that appeared on the back side, the higher the permeability of the artificial leather sample).

In a further investigation about inoculating *E. coli* solutions onto the artificial leather samples (see Figure 1), it was found that the artificial leather samples were more hydrophilic, and the *E. coli* inoculums were permeable from the face to back sides. This can probably be explained by the fact that the Enrichment Device could not collect the *E. coli* inoculums by swabbing properly in the Rapid Test for plastics. Hence, the Rapid Test for textiles was subsequently conducted to examine the artificial leather samples; the results are summarised in Table 6. Compared with the results from the Rapid Test for plastics, the CFU/swab values of the reference setup (direct pipetting to saline solution) and the CAA-treated artificial leather materials showed reasonable results. On the contrary, large amounts of *E. coli* could be cultured from the pristine artificial leather samples inoculated with 1:10^7^ and 1:10^8^ bacterial solutions. These outcomes are crucial in demonstrating the applicability and effectiveness of the test methods of Rapid Tests for porous (i.e., textiles) and non-porous (i.e., plastics) materials. In addition, the biotoxicity concern associated with the PU coating material, which was previously raised owing to false positive antibacterial effects, was addressed. To verify the results of PU artificial leathers from the Rapid Test for textiles, they were further assessed using the AATCC TM-100. Table 7 presents that the test results, which are consistent with those from the Rapid Test for textiles.

## 3. Materials and Methods

### 3.1. Materials

Antibacterial and pristine silicone rubber, HDPE, and 3D-printed resins were commercially available products. Silicone rubber is the product of Jiadi Plastic Product Co., Ltd. (Dongguan, China). HDPE sample pieces were produced by Integrated Waste Solutions Group Holdings Limited (Hong Kong, China). The 3D-printed rigid and tough resins were manufactured by Phrozen Tech Co., Ltd. (Taiwan, China) and Siraya Tech (Los Angeles, CA, USA) respectively. PU artificial leather samples were donated by Dongguan Lizhou Textile Leather Co, Ltd. (Dongguan, China). The 100% cotton canvas (fabric weight: 257 g/m^2^) was treated with CAA and a PU-based textile binder using pad-dry-cure technique. PU artificial leather was fabricated by coating the polyurethane polymer, which was pre-mixed with CAA, onto the face side of polyester fabric. All test specimens were evaluated after being conditioned at 21 ± 2 °C and 65 ± 5% relative humidity for 24 h.

### 3.2. Antibacterial Assessments of Plastic Samples by ISO 22196:2011

The antibacterial plastic samples (length × width: 50 mm × 50 mm; thickness: 2 to 4 mm) were prepared and evaluated for efficacy against *E. coli* based on the ISO 22196:2011 [1]. Three sample pieces of each treated material and six sample pieces of each corresponding pristine material were assessed. The concentration of test inoculum used in this study was 1:10^8^, in parallel with that used in the Rapid Tests.

### 3.3. Antibacterial Assessments of Textile Samples by AATCC TM-100

The antibacterial textile samples (length × width: 50 mm × 25 mm) prepared with and without CAA were evaluated for efficacy against *E. coli* based on the AATCC TM-100 [2]. Three pieces of treated and untreated fabric specimens were evaluated; one piece of fabric specimen was sufficient to absorb 1 mL inoculum in each setup. The concentration of test inoculum used in this study was 1:10^8^, in parallel with that used in the Rapid Tests.

### 3.4. Rapid Test for Plastic Samples

The antibacterial and pristine test samples (length × width: 50 mm × 25 mm; thickness: 2 to 4 mm) were prepared and assessed in the Rapid Test for plastics. Three sample pieces of each treated material and six sample pieces of each corresponding pristine material were assessed. A volume of 15 µL of 1:10^8^ or 30 µL of 1:10^7^ *E. coli* solution was inoculated at the centre of a sterile 90 mm diameter Petri dish. The same volumes of *E. coli* solution were directly pipetted into the Enrichment Device using a micropipette (VITLAB, Bavaria, Germany), for the reference setup of direct pipetting. The test inoculum was covered by the test surface of a piece of plastic sample for 10 min; for the swabbing reference setup, it was not covered and was kept stationary for 10 min.

After 10 min, the test sample was removed from the Petri dish, and the inoculum on the surface of the test sample was collected by the swab of the Enrichment Device. The inoculum on the Petri dish was then swabbed vertically, horizontally, and in both diagonal directions, as in a crisscross pattern, using the same Enrichment Device (Hygiena International, Watford, UK). The subsequent enrichment, incubation, detection procedures, and result interpretations were conducted according to the technical documents from Hygiena International (Watford, UK) [31,32]. However, the incubation time of the Enrichment Devices for the samples inoculated with 1:10^8^ and 1:10^7^ could be reduced to 210 and 300 min, respectively. The incubation time was shorter than the 360 min suggested by Hygiena International. This is because the MicroSnap *E. coli* test system was originally designed for sampling very limited and minimal quantities of *E. coli* from environmental surfaces and pushing the lowest detection level as far downward as possible. The approach of the Rapid Tests involved the evaluation of antimicrobial sample surfaces inoculated with high concentrations of *E. coli* solution. Shorter incubation time was sufficient for detection.

### 3.5. Rapid Test for Textile Samples

The antibacterial textile samples (length × width: 20 mm × 20 mm) prepared with and without CAA were assessed in the Rapid Test for textiles. Three pieces of treated and untreated fabric specimens were evaluated; one piece of fabric specimen was sufficient to absorb the volume of inoculum used in each setup. Controlled volumes (µL) of 1:10^8^, 1:10^7^, and 1:10^6^ *E. coli* solutions were inoculated at the centre of the test specimens placed in a sterile Petri dish. The same volumes of 1:10^8^, 1:10^7^, and 1:10^6^ *E. coli* solutions were directly pipetted into a specified volume of 0.85% (*w*/*v*) saline solution (see Table 8) using a micropipette for the reference setups of direct pipetting. Every inoculated specimen was kept stationary on the Petri dish at room temperature for 5 min, and then transferred into a sterile 50 mL centrifuge tube containing specified volumes of 0.85% (*w*/*v*) saline solution for bacterial extraction by gentle shaking at room temperature for 5 min. 10-fold serial dilution with 0.85% (*w*/*v*) saline solution was conducted for the dilution approach in verification assessment. 2 mL of the aliquot from each specimen’s extract was transferred into a sterile 15 mL centrifuge tube; the swab of the Enrichment Device was immersed into the 2 mL of aliquot within a 15 mL centrifuge tube for 30 s of sampling. The subsequent enrichment, incubation, detection procedures, and result interpretations were conducted according to the technical documents from Hygiena International [31,32]. The incubation time of all Enrichment Devices in the Rapid Test for textiles was 6 h.

## 4. Conclusions

Rapid assessments for the antibacterial activity of plastic and textile products (in other words, non-porous and porous materials) against *E. coli* are developed. The results obtained from the Rapid Tests for plastics and textiles are strongly correlated to those from standard tests, the ISO 22916 and the AATCC TM-100, respectively. Supported by a case study on examining the antibacterial efficacy of PU artificial leather materials with a hydrophilic surface, the application scope of Rapid Tests for plastics and textiles is clearer for practical testing purposes.

It is believed that preliminary, rapid, and highly standard-correlated antimicrobial assessments can effectively facilitate the development of novel antimicrobial materials, in terms of R&D cycle and quality assurance. Antibacterial plastics and textiles are widely utilised across various fields to provide innovative and reliable antimicrobial solutions for infection prevention and control against the quickly mutating diseases nowadays. While this study focuses on rapid assessment of the efficacies of porous and non-porous materials against *E. coli*, antibacterial function is a primary, fundamental, and essential performance requirement of antimicrobial products to be developed. In common practice, antibacterial effects of the samples and prototypes are usually evaluated prior to other antiviral and antifungal assessments. Thus, rapid antibacterial tests can serve as a “kick-off test” to accelerate the innovation and advancement of antimicrobial products, enabling industries to respond more swiftly to emerging threats and market demands.

## Figures and Tables

**Table 1:** CFU/mL values of *E. coli* collected from the surfaces of different plastic samples with and without antimicrobial agents at 0 and 24 h, as determined according to the ISO 22196:2011.

Plastic Substrate	Sample Type	CFU/mL of *E. coli* at Contact Hours	
		0 h	24 h
Silicone rubber	Non-antimicrobial	8.00 × 10^8^	2.47 × 10^9^
	Antimicrobial	0	0
High-density polyethylene	Non-antimicrobial	6.00 × 10^8^	1.52 × 10^9^
	Antimicrobial	0	0
3D-printed rigid plastic	Non-antimicrobial	1.11 × 10^9^	1.80 × 10^9^
	Antimicrobial	0	0
3D-printed tough plastic	Non-antimicrobial	1.12 × 10^9^	1.84 × 10^9^
	Antimicrobial	0	0

**Table 2:** CFU/swab values of *E. coli* in control setups and collected from the surfaces of different plastic samples with and without antimicrobial agents, as obtained using the Rapid Test.

		Concentration of *E. coli* Solution Inoculated			
Plastic Substrate	Swabbed Sample	1:10^7^		1:10^8^	
		RLU	CFU/Swab	RLU	CFU/Swab
Silicone rubber	Direct pipetting	696	>>1.00 × 10^4^	1525	>>1.00 × 10^4^
	Swabbing	1251	>>1.00 × 10^4^	1670	>>1.00 × 10^4^
	Non-antimicrobial	1763	>>1.00 × 10^4^	2926	>>1.00 × 10^4^
	Antimicrobial	1	<5	5	<15
High-density polyethylene	Direct pipetting	1747	>>1.00 × 10^4^	1402	>>1.00 × 10^4^
	Swabbing	2735	>>1.00 × 10^4^	715	>>1.00 × 10^4^
	Non-antimicrobial	2113	>>1.00 × 10^4^	753	>>1.00 × 10^4^
	Antimicrobial	0.3	<5	0	<1
3D-printed rigid plastic	Direct pipetting	440	>>1.00 × 10^4^	1746	>>1.00 × 10^4^
	Swabbing	1357	>>1.00 × 10^4^	3048	>>1.00 × 10^4^
	Non-antimicrobial	1244	>>1.00 × 10^4^	3404	>>1.00 × 10^4^
	Antimicrobial	0	<1	5	<35
3D-printed tough plastic	Direct pipetting	1584	>>1.00 × 10^4^	209	>>1.00 × 10^4^
	Swabbing	269	>>1.00 × 10^4^	1445	>>1.00 × 10^4^
	Non-antimicrobial	1989	>>1.00 × 10^4^	2224	>>1.00 × 10^4^
	Antimicrobial	0	<1	0	<1

**Table 3:** CFU/mL values of *E. coli* collected from cotton fabrics with 0%, 0.5%, and 1.5% (*w*/*w*) CAA at 0 and 24 h, determined according to AATCC TM 100-2019.

CAA % (*w*/*w*) on Cotton Fabrics	CFU/mL of *E. coli* Collected at Different Hours	
	0 h	24 h
0	9.08 × 10^8^	3.50 × 10^9^
0.5	0	0
1.5	0	0

**Table 4:** CFU/swab values of *E. coli* in control setup and collected from the cotton samples with different % of CAA (*w*/*w*), as obtained using the Rapid Test for textiles with a volume approach.

	Concentration of *E. coli* Solution								
	1:10^8^			1:10^7^			1:10^6^		
Volume of *E. coli* Solution (µL)	20.1	15.1	10.1	30.1	20.1	10.1	30.1	20.1	10.1
Swab Sample	RLU (CFU/Swab)	RLU (CFU/Swab)	RLU (CFU/Swab)	RLU (CFU/Swab)	RLU (CFU/Swab)	RLU (CFU/Swab)	RLU (CFU/Swab)	RLU (CFU/Swab)	RLU (CFU/Swab)
Direct pipetting to saline solution #	5551 (>>1.00 × 10^4^)	4007 (>>1.00 × 10^4^)	4491 (>>1.00 × 10^4^)	3500 (>>1.00 × 10^4^)	4747 (>>1.00 × 10^4^)	4245 (>>1.00 × 10^4^)	1386 (>>1.00 × 10^4^)	730 (>>1.00 × 10^4^)	180 (<5100)
Pristine cotton fabric	4602 (>>1.00 × 10^4^)	3855 (>>1.00 × 10^4^)	3532 (>>1.00 × 10^4^)	3355 (>>1.00 × 10^4^)	3113 (>>1.00 × 10^4^)	1887 (>>1.00 × 10^4^)	548 (>>1.00 × 10^4^)	15 (<140)	1 (<5)
Cotton fabric with 0.5% CAA	0 (<1)	2 (10)	0 (<1)	0 (<1)	1 (<5)	3 (<15)	0 (<1)	0 (<1)	0 (<1)
Cotton fabric with 1.5% CAA	0 (<1)	0 (<1)	1 (<5)	0 (<1)	1 (<5)	1 (<5)	0 (<1)	0 (<1)	0 (<1)

# 0.85% (*w*/*v*) saline solution: RLU = 1; estimated CFU/swab = <5.

**Table 5:** CFU/swab values of *E. coli* in control setup and collected from the cotton samples with different % of CAA (*w*/*w*), as obtained using the Rapid Test for textiles with a dilution approach.

	Concentration of *E. coli* Solution (Volume of Inoculum)								
	1:10^8^ (20.1 µL)			1:10^7^ (30.1 µL)			1:10^6^ (30.1 µL)		
Dilution Factor	1	10^−1^	10^−2^	1	10^−1^	10^−2^	1	10^−1^	10^−2^
Swab Sample	RLU (CFU/Swab)	RLU (CFU/Swab)	RLU (CFU/Swab)	RLU (CFU/Swab)	RLU (CFU/Swab)	RLU (CFU/Swab)	RLU (CFU/Swab)	RLU (CFU/Swab)	RLU (CFU/Swab)
Direct pipetting to saline solution #	5255 (>>1.00 × 10^4^)	4167 (>>1.00 × 10^4^)	32 (<450)	3862 (>>1.00 × 10^4^)	513 (>>1.00 × 10^4^)	3 (<15)	133 (<3230)	0 (<1)	0 (<1)
Pristine cotton fabric	3206 (>>1.00 × 10^4^)	2652 (>>1.00 × 10^4^)	20 (<200)	3803 (>>1.00 × 10^4^)	34 (<480)	2 (<10)	4 (<20)	1 (<5)	2 (<10)
Cotton fabric with 0.5% CAA	0 (<1)	2 (<10)	2 (<10)	1 (<5)	1 (<5)	1 (<5)	2 (<10)	1 (<5)	1 (<5)
Cotton fabric with 1.5% CAA	1 (<5)	0 (<1)	0 (<5)	1 (<5)	3 (<15)	1 (<5)	0 (<1)	0 (<1)	0 (<1)

# 0.85% (*w*/*v*) saline solution: RLU = 1; Estimated CFU/swab = <5.

**Table 6:** CFU/swab values of *E. coli* in control setup and collected from the PU samples with different % of CAA (*w*/*w*), as obtained using the Rapid Tests for plastics and textiles.

Concentration and Volume of *E. coli* Solution	Rapid Test for Plastics		Rapid Test for Textiles #	
	1:10^7^ (30.1 µL)	1:10^8^ (20.1 µL)	1:10^7^ (30.1 µL)	1:10^8^ (20.1 µL)
	RLU (CFU/Swab)	RLU (CFU/Swab)	RLU (CFU/Swab)	RLU (CFU/Swab)
Direct pipetting @	893 (>>1.00 × 10^4^)	2386 (>>1.00 × 10^4^)	3608 (>>1.00 × 10^4^)	3203 (>>1.00 × 10^4^)
Swabbing	1607 (>>1.00 × 10^4^)	5076 (>>1.00 × 10^4^)	N.A.	N.A.
PU with 0% CAA	2 (<10)	1 (<5)	2190 (>>1.00 × 10^4^)	4754 (>>1.00 × 10^4^)
PU with 1% CAA	1 (<5)	0 (<1)	0 (<1)	5 (<30)
PU with 2% CAA	2 (<10)	0 (<1)	1.3 (<10)	3.7 (<20)

# saline solution: RLU = 0; Estimated CFU/swab = <1; @ for plastic test: direct pipetting inoculum to Enrichment Device; for textile test: direct pipetting inoculum to 0.85% (*w*/*v*) saline solution and collected by Enrichment Device.

**Table 7:** CFU/mL values of *E. coli* collected from PU samples with different % of CAA (*w*/*w*) at 0 and 24 h, determined according to the AATCC TM 100-2019.

CAA% (*w*/*w*) on PU Leathers	CFU/mL of *E. coli* Collected at Different Hours	
	0 h	24 h
0	4.20 × 10^8^	6.40 × 10^8^
1	0	0
2	0	0

**Table 8:** Volumes of saline solution used for extracting different volumes of *E. coli* inoculums from textile test specimens.

Volume of *E. coli* Inoculum (µL)	Volume of 0.85% (*w*/*v*) Saline Solution (mL)
30.1	2.98
20.1	2.99
15.1	2.99
10.1	3.00

## Data Availability

The data presented in this study are available on request from the corresponding author (privacy and legal).
